# Rhythm, reading, and sound processing in the brain in preschool children

**DOI:** 10.1038/s41539-021-00097-5

**Published:** 2021-06-29

**Authors:** Silvia Bonacina, Stephanie Huang, Travis White-Schwoch, Jennifer Krizman, Trent Nicol, Nina Kraus

**Affiliations:** 1 Auditory Neuroscience Laboratory, Northwestern University, Evanston, IL USA; 2 Department of Communication Sciences and Disorders, Northwestern University, Evanston, IL USA; 3 Department of Neurobiology, Northwestern University, Evanston, IL USA

**Keywords:** Sensorimotor processing, Auditory system, Dyslexia

## Abstract

A child’s success in school relies on their ability to quickly grasp language and reading skills, the foundations of which are acquired even before entering a formal classroom setting. Previous studies in preschoolers have begun to establish relationships linking beat synchronization, preliteracy skills, and auditory processing. Beat synchronization involves the integration of sensorimotor systems with auditory and cognitive circuits and, therefore calls on many of the same neural networks as language. Using a drumming task, we analyzed the relationship between children’s ability to maintain an isochronous beat with preliteracy skills and frequency following responses (FFRs) in over 150 preschoolers. We show that preschoolers who performed well on the beat synchronization task outscored their peers on all preliteracy measures and had more robust FFRs. Furthermore, the good synchronizers experienced less degradation of certain FFR measures when listening in noise. Together, our results are consistent with the view that rhythm, preliteracy, and auditory processing are interconnected during early childhood.

## Introduction

Beat synchronization, a task requiring precise integration of auditory perception and motor production, has offered an interesting window into the biology of reading ability and its substrate skills. Beat synchronization performance varies widely among young children, with poorer performers also often struggling with reading development. The origin of this struggle is still unclear, and it is thought to reflect either a primary sensory deficit in auditory rhythm perception that in turn affects the temporal precision of action, an independent deficit related to the developing motor system, or both. A study tried to pinpoint the origin by comparing children with dyslexia and typically developing children on beat-related steady-state evoked potentials in three conditions: (i) when passively listening to a beat, (ii) when tapping with the right hand, and (iii) when tapping with the left hand to a metronome pulse. The data documented atypical neural entrainment to the beat and greater neural power in the passive listening condition for children with dyslexia, without any difference between the groups for tapping performance alone, supporting the hypothesis of an auditory rhythm perception deficit in developmental dyslexia that affects temporal precision of action (Colling, Noble, and Goswami, 2017^1^). This finding extends support for the Temporal Sampling Framework (TSF) hypothesis which, by now, has offered solutions to many controversies among competing theories of dyslexia. The TSF explains the auditory sensory difficulties associated with dyslexia by an oscillatory perspective with inefficiency in the tracking of low-frequency modulation (1.5–10 Hz), such as the amplitude envelope onset (rise time) of sounds. Our group in the past provided further support for the TSF hypothesis by showing, in preschoolers, how the precision of neural encoding of the envelope of the speech syllable can be explained by both phonological and beat synchronization skills (Carr et al., 2014^2^).

In fact, the interrelationship among beat synchronization skills, neural processing of sound, and phonological skills has been documented across age groups using different methodologies (Huss, Verney, Forsker, Mead, Goswami, 2011^3^; Carr, 2014^2^; 2016^4^; Colling, Noble, & Goswami, 2017^1^; Tierney and Kraus, 2013^5^; Tierney, White-Schwoch, MacLean, and Kraus, 2017^6^).

Our group explored these relationships by collecting frequency-following responses (FFRs) in preschool-aged children who varied in their synchronization ability. The FFR is a measure of synchronous sound-evoked neural activity arising predominately from the inferior colliculus (IC) of the auditory midbrain that faithfully reproduces both spectral and temporal stimulus features. In fact, even though there have been hints of the idea that FFR relies on a mix of subcortical nuclei with potential cortical contribution depending on the recording techniques, stimulus, and participant demographics (Coffey et al., 2019^7^), we have strong reasons to believe the FFR yields a subcortical response (White-Schwoch, Krizman, Nicol, and Kraus, 2021^8^; White-Schwoch et al., 2017^9^). Thanks to its unique complexity and richness, FFR provides various measures that can be analyzed to explore timing (i.e., peak latencies, phase-locking consistency), magnitude (i.e., RMS amplitude, signal-to-noise ratio, and frequency encoding), and fidelity of a response (i.e., envelope encoding precision, stimulus to response correlation, and response consistency) (Krizman and Kraus, 2019^10^). By using the FFR, we have a window into encoding of these discrete response components to explore whether differences in encoding of temporal cues in the speech sound can account for variability in synchronization ability and language development.

Preschoolers represent a particularly interesting sample to study with respect to the interrelationship among beat synchronization, neural processing of sound, and phonological skills, both theoretically and clinically. Theoretically, they are at an age of constant developmental changes, meaning that their brain plasticity and “immaturity” is molded over time by genetics and experience. Clinically, there is an urgency of identifying reading struggles as early as possible, even before a child begins learning to read. With respect to this age group, we previously reported positive relationships between beat synchronization and preliteracy skills, namely phonological awareness, auditory short-term memory, and rapid naming in a group of 35 children between the ages of 3 and 5 years (Carr et al., 2014^2^). Furthermore, children’s performance on the synchronization task correlated positively with several measures of the FFR.

The above-mentioned study on preschoolers (Carr et al., 2014^2^) compared a range of synchronization ability (“Synchronizers” vs. “Non-synchronizers”) and reported an association between children’s synchronization skills and a measure of the precision of neural encoding of the speech envelope evoked collected by the FFR. Following these initial findings, a second study focused specifically on the relationships between beat synchronization consistency and other FFR measures indicative of the consistency of the neural response to sound within the IC. This study was confined to a subset of 25 children who passed a selection criterion for their ability to synchronize motor movements to isochronous beats (Carr et al., 2016^4^). Within this group, there were systematic correlations between the ability to synchronize to a beat and two other measures from the FFR-response consistency and phase-locking consistency.

Those studies first identified associations among rhythm ability, sound processing in the brain, and preliteracy skills, and suggest the potential use of nonverbal behavioral test of rhythmic ability possibly combined with the objective neural metric collected by the FFR to detect reading strugglers as early as 3 years of age. However, those studies are characterized by a relatively small dataset and are mostly devoted to simple listening conditions, such as listening in quiet (Carr et al., 2014^2^ considered the Noise presentation mode more as control than as an independent variable), and robust FFR response components (i.e., the added polarity). The first element prevents to capture of enough natural variability in developing children in their age, socioeconomic status, musical ability, and other demographic factors. The second choice does not consider that in real life, listening often occurs in complex environments (e.g., noise), and that higher frequency encoding highlighted in the subtracted responses is informative of phoneme identification, crucial in the reading-learning process.

In the current study, we have the uncommon opportunity to thoroughly explore the same relationships between beat synchronization, FFR measures, and preliteracy skills among a large, diversely representative sample (*N* > 150) of preschoolers adding a more thorough consideration of the impact of processing challenging stimulus conditions in all their different factors (i.e., stimulus polarity, time region, …) at a neural level.

The aim of this study is to firmly establish the connections between beat synchronization, auditory processing, and preliteracy skills in a large group of preschoolers. We predict that preschoolers who perform better on a beat synchronization task will also score higher than preschoolers who are poor synchronizers on preliteracy skill tasks and will also have more precise auditory neural responses to speech sounds.

## Results

### “Synchronizers” outperform “Non-synchronizers” on preliteracy skills and music discrimination task

**Fig. 1:**
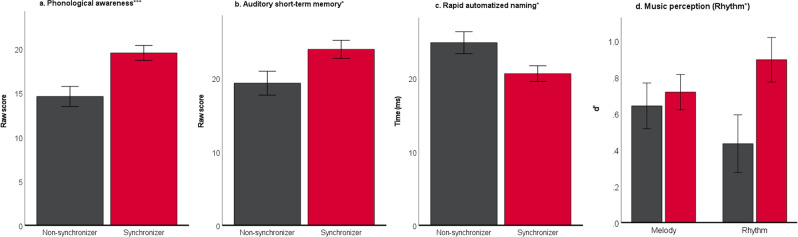
Synchronizers (red) perform better than Non-synchronizers (dark gray) on all preliteracy skill tasks. One-way ANOVAs were used to compare mean performances between the two synchronization groups, Non-synchronizers, and Synchronizers, on preliteracy tasks of (**a**) phonological awareness (*F* = 11.680, *p* < 0.001, η*p*^2^ = 0.175), (**b**) auditory short-term memory (*F* = 4.773, *p* = 0.031, η*p*^2^ = 0.050), (**c**) rapid automatized naming of objects and colors (*F* = 4.881, *p* = 0.030, η*p*^2^ = 0.053), and (**d**) music perception (significant difference was found for the rhythm subtest (*F* = 5.083, *p* = 0.027, η*p*^2^ = 0.052), but not for the melody subtest. Significant differences reveal Synchronizers scoring higher on all of the tasks than Non-synchronizers. (*p < 0.05, **p < 0.01, ***p < 0.005; Error bars at ± 1 standard error).

“Synchronizers” scored higher than “Non-synchronizers” on all of the preliteracy skills that were evaluated. The preliteracy skills included phonological awareness (*F*_(1,55)_ = 11.680, *p* < 0.001, η*p*^2^ = 0.175), auditory short-term memory (*F*_(1,91)_ = 4.773, *p* = 0.031, η*p*^2^ = 0.050), and rapid automatized naming across both objects and colors (*F*_(1,88)_ = 4.881, *p* = 0.030, η*p*^2^ = 0.053). As for the music perception task, “Synchronizers” scored higher than “Non-Synchronizers” for the Rhythm subtest (*F*_(1,94)_ = 5.083, *p* = 0.027, η*p*^2^ = 0.52), but not for the Melody subtest (*F*_(1,95)_ = 0.177, *p* = 0.675, η*p*^2^ = 0.002) (Fig. 1).

Descriptive statistics for all preliteracy and musical perception measures for “Synchronizers” and “Non-synchronizers” are reported in Table 1.

**Table 1:** Descriptive statistics (Mean, SD) for preliteracy and musical perception measures organized by synchronization group.

Neuropsychological assessment (preliteracy and music perception)	Non-synchronizers	Synchronizers
	(M, SD)	(M, SD)
Phonological awareness (raw score)	14.95, 5.84 (*N* = 21)	19.35, 4.70 (*N* = 37)
Auditory short-term memory (raw score)	19.80, 10.12 (*N* = 35)	23.68, 8.59 (*N* = 59)
Rapid automatized naming (milliseconds)		
- Colors	26.32, 13.20 (*N* = 32)	21.29, 10.10 (*N* = 59)
- Objects	22.83, 7.90 (*N* = 32)	20.28, 5.72 (*N* = 59)
Gordon music perception (d’ prime)		
- Melody	0.63, 0.68 (*N* = 36)	0.73, 0.75 (*N* = 59)
- Rhythm	0.51, 1.00 (*N* = 36)	0.84, 0.90 (*N* = 59)

### “Synchronizers” exhibit more precise encoding of the speech envelope than “Non-synchronizers”

**Fig. 2:**
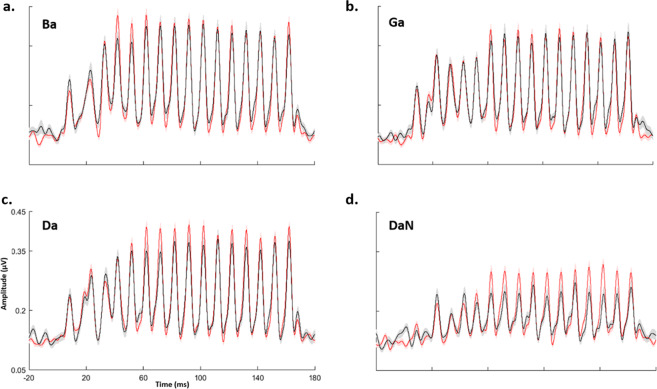
Envelope response to the four speech stimuli compared between Non-synchronizers (black) and Synchronizers (red). Envelope responses were averaged across participants in the Non-Synchronizer group (*N* = 37) and the Synchronizer group (*N* = 63). The averages for each stimulus ((**a**) Ba; (**b**) Ga; (**c**) Da; (**d**) DaN) were then plotted in amplitude versus time graph. Solid lines represent means and shaded lines represent ± 1 standard error.

**Fig. 3:**
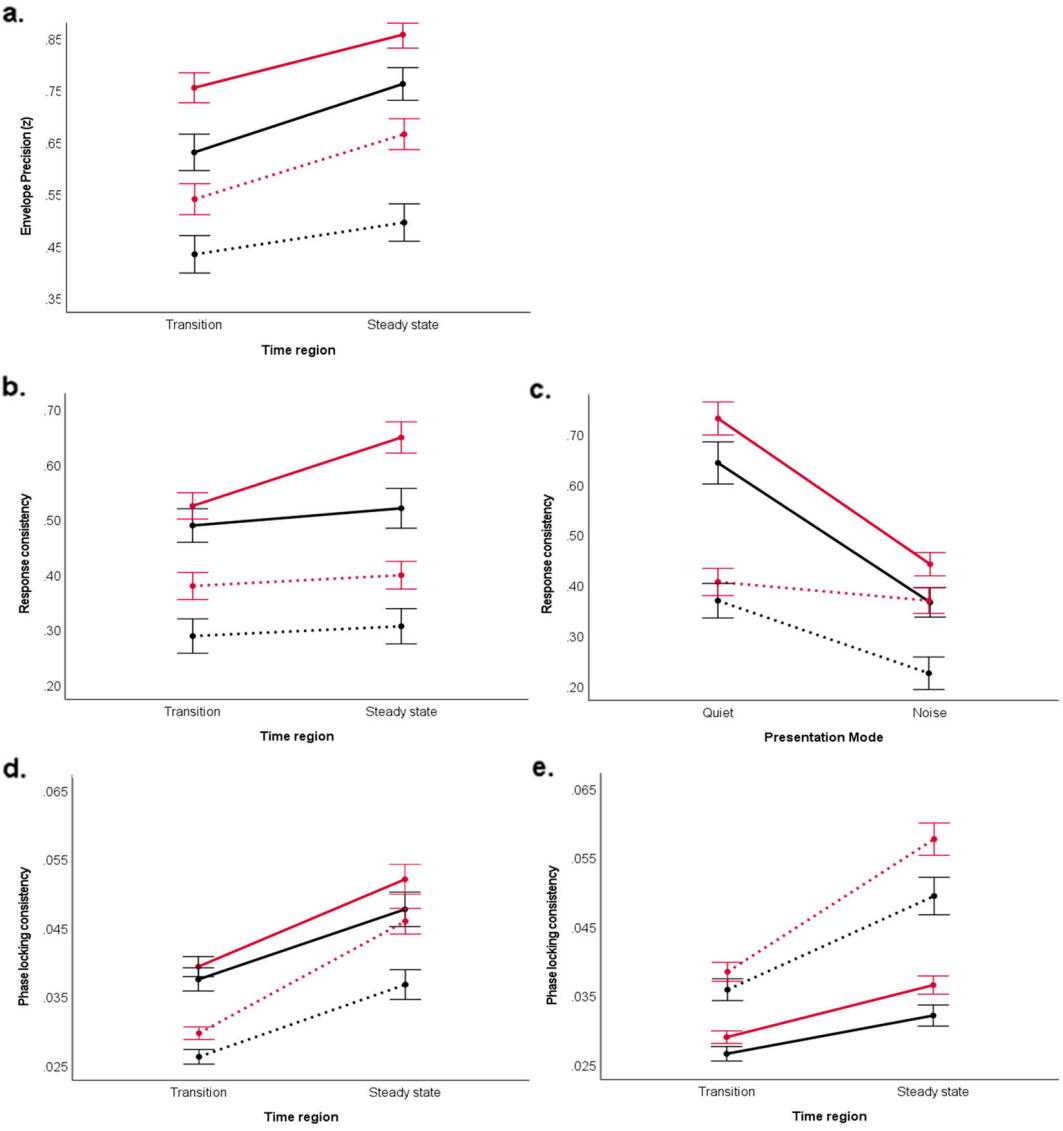
Interaction between presentation mode, time region of the FFR, frequency, and synchronization ability group (Non-synchronizer: black, Synchronizer: red) in determining envelope-encoding precision, response consistency, and phase-locking consistency. A repeated-measures ANOVA was used to evaluate the effects of the between-group variable (synchronization ability group) with the within-group variables considered. Significant interaction effects are represented. (**a**) As for envelope-encoding precision, a significant interaction was found between all three factors-presentation mode (Quiet: solid line; Noise: dotted line) x time region x synchronization group (*F* = 4.314, *p* = 0.012, η*p*2 = 0.071). As for response consistency, the following significant interactions are represented: (**b**) polarity (Added: solid line; Subtracted: dotted line), time region, and synchronization group (*p* < 0.01). (**c**) Presentation mode, polarity (Added: solid line; Subtracted: dotted line), and synchronization group (*p* < 0.01). As for phase-locking consistency, the following significant interactions are represented: (**d**) time region of the FFR, presentation mode (Quiet: solid line; Noise: dotted line), and synchronization group (*p* < 0.05), and (**e**) an interaction between time region of the FFR, frequency (Low: solid line; High: dotted line), and synchronization group (*p* < 0.05). For all graphs, Non-synchronizer means are shown by the black lines and Synchronizer means are the red lines. Means for the FFR measure considered are displayed on the *y*-axis. Error bars represent ± 1 standard error.

Across all four stimuli ([ba], [da], [ga], and [da]noise), the main effect of synchronization group was found, with the “Synchronizers” showing greater envelope precision than “Non-synchronizers” (*F*_(1, 87)_ = 10.860, *p* = 0.001, η*p*^2^ = 0.111) (Fig. 2). Furthermore, collapsing across synchronization group, envelope precision was also found to be higher among the quiet stimuli than the noisy stimulus (*F*_(1, 87)_ = 10.867, *p* = 0.001, η*p*^2^ = 0.111). Envelope precision is also weaker in the formant transition but stronger in the steady-state portion of the response (*F*_(1, 87)_ = 6.602, *p* = 0.012, η*p*^2^ = 0.071). An interaction was found between all the three conditions group by stimulus by time region (*F*_(1, 87)_ = 4.314, *p* = 0.041, η*p*^2^ = 0.047) (Fig. 3a).

### “Synchronizers” have more consistent FFRs than “Non-synchronizers”

**Fig. 4:**
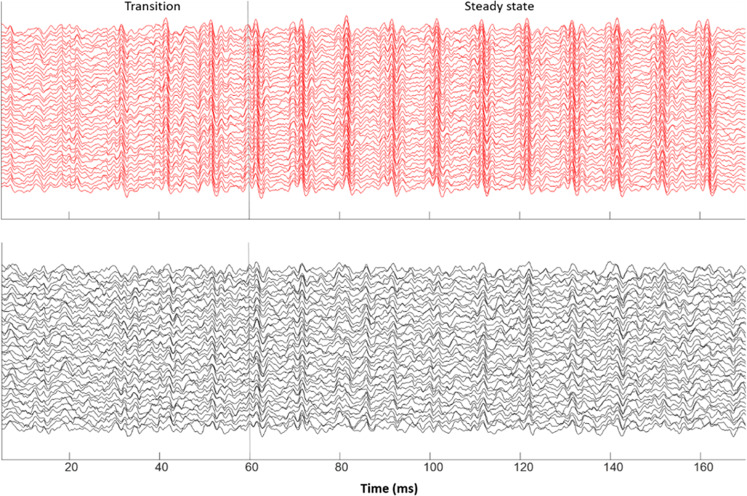
Visual representation of the difference in neural stability seen between FFRs from a representative Synchronizer (red) versus a representative Non-synchronizer (black). In each panel, forty samples of 2000-sweep subaverages are overlaid, giving a visual indicator of what it means to have high or low neural stability of the FFR. The responses shown were elicited from a [ba] stimulus, presented in a quiet condition.

Regardless of stimulus, “Synchronizers” had higher response consistency (i.e., more consistent neural responses between trials) than “Non-synchronizers” (*F*_(1,79)_ = 4.593, *p* = 0.035, η*p*^2^ = 0.055). Consider Fig. 4 for a visual representation of the difference in neural stability seen between FFRs from a representative Synchronizer versus a representative Non-synchronizer. Furthermore, when collapsing across the synchronization group, we observed that participants had higher RC with the quiet stimuli than with the noisy stimulus (*F*_(1,79)_ = 12.545, *p* = 0.001, η*p*^2^ = 0.137). However, RC was not found to be significantly lower in the formant transition region than the steady-state vowel time region (*F*_(1,79)_ = 0.879, *p* = 0.351, η*p*^2^ = 0.011) as was seen with envelope precision. For response consistency, we also introduced stimulus polarity as one of the within-group variables into the repeated-measures ANOVA. We found that RC was higher in the added polarity than subtracted (*F*_(1,79)_ = 23.197, *p* < 0.001, η*p*^2^ = 0.227).

There were several significant interactions found between the variables we analyzed. An interaction was found between the synchronization group and time region of the FFR (*F*_(1,79)_ = 8.091, *p* = 0.006, n*p*^2^ = 0.093) While both “Non-synchronizers” and “Synchronizers” had higher RC in the steady-state than the transition region, Synchronizers showed a larger difference in RC between the two time regions. Other interactions between 3 variables included one between synchronization group, mode of presentation, and polarity (*F*_(1,79)_ = 9.860, *p* = 0.002, n*p*^2^ = 0.111) and another between synchronization group, time region, and polarity (*F*_(1,79)_ = 4.316, *p* = 0.041, n*p*^2^ = 0.052) (Fig. 3b and c). Both of these interactions showed a different pattern between the synchronization groups: first, the “synchronizers” response in the added polarity shows a larger enhancement in RC between the two time regions compared with the “Non-synchronizers”; second, “synchronizers” response in the subtracted polarity show a smaller change in RC between the two presentation modes compared with the “Non-synchronizers” ones. Last, there was also an interaction between all four of the variables analyzed for RC, synchronization group by mode of presentation by time region by polarity (*F*_(1,79)_ = 11.068, *p* = .001, n*p*^2^ = .123).

### “Synchronizers” have higher phase-locking consistency (PLC) than “Non-synchronizers”

**Fig. 5:**
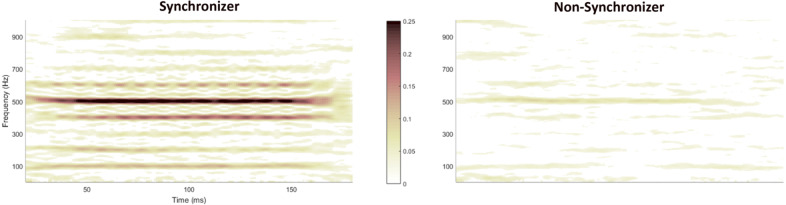
Visual representation of the difference in phase-locking consistency between a representative Synchronizer (left) versus a representative Non-synchronizer (right). The responses shown were elicited from a [ba] stimulus, presented in a quiet condition. Darker colors depict higher phase-locking consistency. The PLF is calculated on overlapping 40-ms bins in the response and is illustrated on a colorscale, ranging from yellow (0, complete phase variability) to red (0.25, higher phase consistency).

When comparing the averages between the two groups, “Synchronizers” display greater phase-locking consistency than “Non-synchronizers” (F_(1,76)_ = 4.498, *p* = 0.037, η*p*^2^ = 0.056). Consider Fig. 5 for a visual representation of the difference in phase-locking consistency between a representative Synchronizer versus a representative Non-synchronizer. The PLC trends relating to the mode of presentation and time region of the FFR are consistent with what was found with envelope precision and response consistency. Regardless of beat synchronization, the children have higher PLC to the quiet stimulus than with the noisy stimulus (*F*_(1,76)_ = 8.927, *p* = 0.004, η*p*^2^ = 0.105), and they also have lower PLC in the formant transition compared with the steady-state (*F*_(1,76)_ = 22.238, *p* < 0.0005, η*p*^2^ = 0.226). Contrary to what was seen with RC, there is no significant effect of added or subtracted polarity on PLC (*F*_(1,76)_ = 0.559, *p* = 0.457, η*p*^2^ = 0.007). For PLC, we introduced frequency ranges as another independent variable and found that participants exhibited greater PLC in the higher frequency range (500–1000 Hz) than the lower frequency range (100–400 Hz) (*F*_(1,76)_ = 10.585, *p* = 0.002, η*p*^2^ = 0.122).

Significant interactions are listed as follows: 1) between synchronization group and the time region (F_(1,76)_ = 9.182, *p* = 0.003, np^2^ = 0.108) where “Synchronizers” show a greater increase in PLC than “Non-synchronizers” when looking at this measure from the transition to the steady-state, 2) between synchronization group, time region, and the presentation mode (*F*_(1,76)_ = 5.217, *p* = 0.025, n*p*^2^ = 0.064), and 3) between synchronization group, time region, and frequency (*F*_(1,76)_ = 7.037, *p* = 0.010, n*p*^2^ = 0.085) with “Synchronizers” showing a larger change in PLC between the two time regions when dealing with a noisy stimulus and with respect to high frequency than “Non-synchronizers” (Fig. 3d and e).

### Spotlight on the “Synchronizers-at-one-rate-only”

In total 56 (26 F) children belong to this group (Mean age = 3.8; SD = 0.49).

This subgroup represents a fascinating sample among the children included in this study. Among these children, 31 were deemed “Synchronizers” at the 600 ISI only and 25 children were “Synchronizers” at the 400 ISI only. Mean age comparisons documented that the kids in this group are significantly younger than the ones in the other two groups (by about 0.4 years, *p* = 0.001). This piece of information is illustrative of something unique of this group and supports our uncertainty on how to interpret their rhythmic performance. Therefore, we have devoted the present section to exploring their performance across both the behavioral and FFR data considered with respect to the other two groups.

**Fig. 6:**
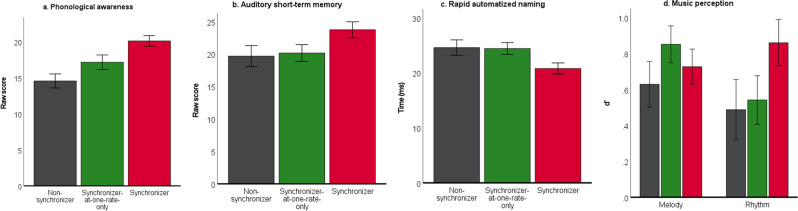
Synchronizers (red), Synchronizers only at one rate (green), and Non-synchronizers (dark gray) performance across preliteracy and music discrimination tasks. (**a**) Phonological awareness; (**b**) Auditory short-term memory; (**c**) Rapid automatized naming; (**d**) Music perception.

Even though from a statistical perspective, the behavioral data did not show significant differences between the “Synchronizers-at-one-rate-only” group and the other two groups (pairwise comparisons revealed meaningful differences only for Phonological awareness (trending, *p* = 0.064) and Rapid automatized naming (*p* = 0.004) between “Synchronizers-at-one-rate-only” and “Synchronizers”), by looking at Fig. 6, it is possible to appreciate how the “Synchronizers-at-one-rate-only” group qualitatively fits between the Non-synchronizer and the Synchronizer groups across the four tests considered.

**Fig. 7:**
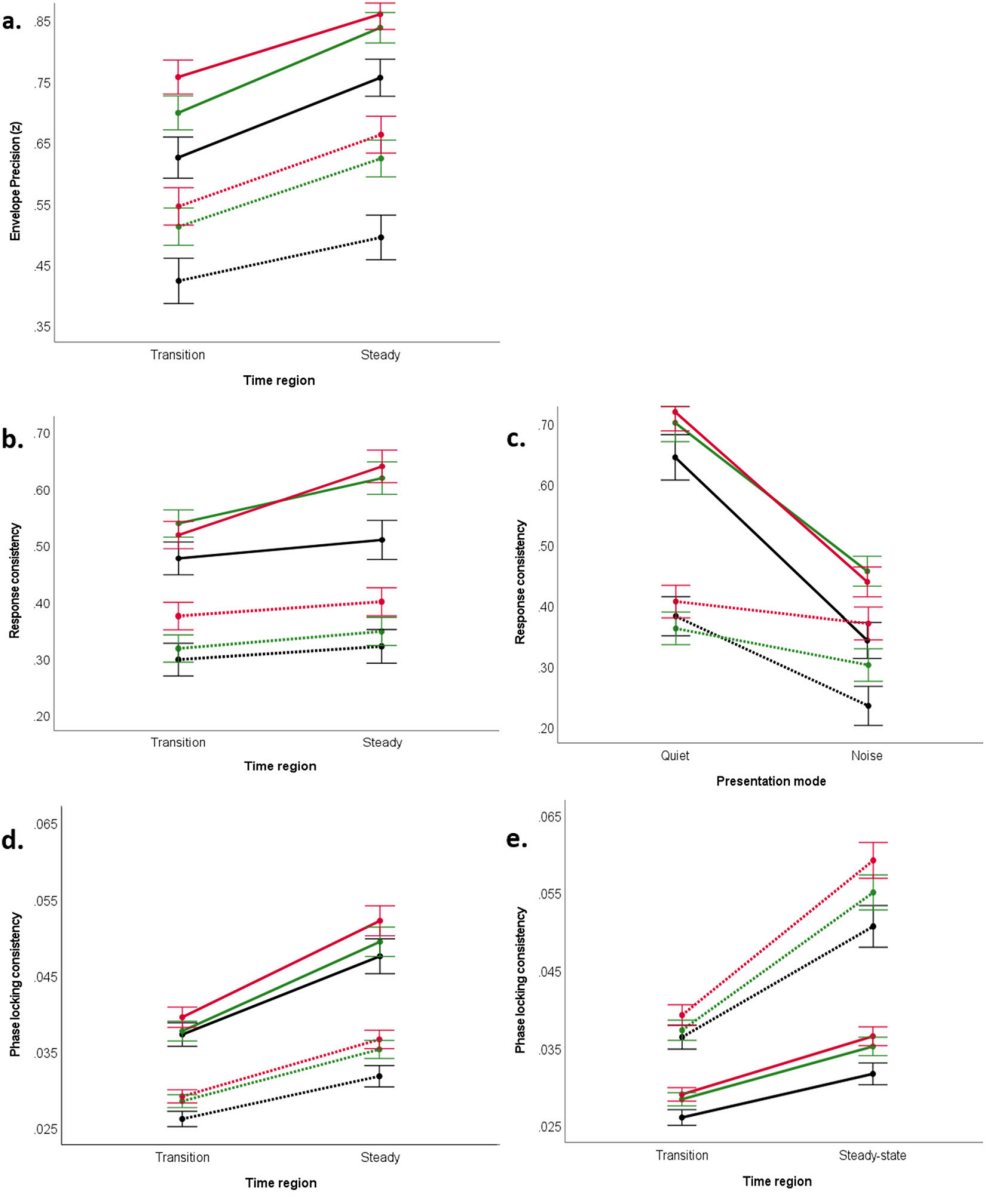
Interaction between presentation mode, time region of the FFR, frequency, and synchronization ability group (Non-synchronizer: dark gray, Synchronizer: red, Synchronizer at one rate only: green) in determining envelope-encoding precision, response consistency, phase-locking consistency. (**a**) Envelope-encoding precision, Quiet: solid line - Noise: dotted line; (**b**, **c**) response consistency, Added: solid line-Subtracted: dotted line; (**d**) phase-locking consistency, Quiet: solid line-Noise: dotted line; (**e**) phase-locking consistency, Low frequency: solid line; High frequency: dotted line.

As for the FFR data, pairwise comparisons documented significant differences between the “Synchronizers-at-one-rate-only” group and the other two groups only for envelope-encoding precision (*p* = 0.036). By looking at Fig. 7, it is possible to appreciate where the “Synchronizers-at-one-rate-only” group fits across the several FFR components and all the different levels we consider for our main analysis.

Next, we compared children who were Synchronizers at the 400 ISI with those who were Synchronizers at the 600 ISI across all the measures considered. The behavioral data documented only a trending difference between the groups for rapid automatized naming (*p* = 0.059) with Synchronizers at 600 ISI only performing better than the other group.

As for the FFR data, three separate RM Anova were performed across the three FFR measures considered and their respective levels. A significant difference between the groups was revealed only for the envelope-encoding precision with a better response for Synchronizers at 600 ISI only (*F*_(1,49)_ = 4.507, *p* = 0.039, n*p*^2^ = 0.084) across the different within-subject factors considered.

From a statistical perspective, these data do not reveal any precise patterns. The last analyses are possibly suggestive of a more similarity between Synchronizers at 600 ISI only with the “Synchronizers”. However, they call for further investigation. Longitudinal analysis may clarify whether the children within this group will evolve into “Synchronizers”, will persist in their good performance only at one rate, or will worsen.

## Discussion

Using a beat synchronization task to measure children’s rhythm ability, we found that preschoolers who performed well on the rhythm task (i.e., those who were able to drum consistently to two rates of presented beats, Synchronizers) scored higher than children who drummed inconsistently (Non-synchronizers) on measures of phonological awareness, auditory short-term memory, rapid naming of both colors and objects, and musical rhythm discrimination. When comparing the frequency-following responses between the two synchronization groups, Synchronizers had higher measures of envelope-encoding precision, response consistency, and phase-locking consistency than Non-synchronizers.

The acquisition of reading skills depends upon integrating what is processed by the visual system with neural circuits that underlie the comprehension of spoken language. Previous research has emphasized how a child’s ability to perceive and entrain to timing cues aids first with speech, then with reading development (White-Schwoch et al., 2015^11–13^). Evidence from these studies has pointed particularly to subcortical neural synchrony and speech-envelope tracking as neural measures that depended upon reading fluency and comprehension (Carr et al., 2014^2^; 2016^4^).

Here we established relationships between rhythm ability, preliteracy skills, and frequency-following responses in a typically developing population of preschoolers, aged 3–5 years old. Keeping in mind the role of rhythm on language (Falk, 2004^14^; DeLong, Urbach, and Kutas, 2005^15^; Slater et al., 2018^16^; Snowling et al., 2018^17^; Lagrois, Palmer, and Peretz, 2019^18^), we hypothesized there would be a measurable difference in the performance on preliteracy skill tasks and measures of auditory processing as a function of a child’s rhythm ability. Specifically, we predicted that children who were better at rhythm tasks would also have a better grasp of preliteracy skills and have more robust neural responses. Overall, our findings support these hypotheses and predictions-a child’s ability to synchronize to a beat is mirrored in their preliteracy skills and robustness of their auditory midbrain response to speech syllables.

We are able to uphold the same relationships between rhythm, preliteracy, and auditory processing previously detected in studies of ∼20–30 participants in our study of about 100 preschoolers (Table 2 provides a summary of tests and results across studies). In line with Carr et al., 2014^2^, here we corroborate differences between Synchronizers and Non-synchronizers across preliteracy skills and subcortical measures of envelope-encoding precision. With respect to Carr et al., 2016^4^, here we approached the analysis in a different and more comprehensive way without keeping the focus only on Synchronizers, but instead continuing the comparison between Synchronizers and Non-synchronizers across subcortical measures of response consistency and phase-locking consistency. Notably, we extended the relationships between the latter two FFR measures, including the Da stimulus presented with a noisy background.

**Table 2:** Summary table of test and results.

	Measure of…	Carr et al. 2014	Carr et al. 2016	Current study
**WPPSI**	Verbal and Non verbal intelligence	Group difference not sig.	n/a	Group difference not sig.
**CELF P2**	Phonological awareness	**	n/a	***
**CELF P2**	Auditory Short-term Memory	*	n/a	*
**RAN**	Rapid Automatized Naming	**	n/a	*
**Gordon’s AUDIE**	- Melody discrimination	*	n/a	Group difference not sig.
	- Rhythm discrimination	*		*
**FFR**	- Envelope encoding precision	Ba, Ga, Da, DaN **	Ba, Ga, Da ***	Ba, Ga, Da, DaN **
	- Response consistency		Ba, Ga, Da ***	Ba, Ga, Da, DaN *
	- Phase locking consistency			Ba, Ga, Da, DaN *

*indicates presence of significant results (group differences or correlations) reported in Carr et al., 2014; 2016, and in the current study.

The accuracy of neural encoding is dependent on *how challenging the various aspects of the stimulus are to encode*. The fidelity of the FFR to the stimulus is largely dependent on that child’s synchronization ability. Challenging conditions include, for instance, the formant transition period of the FFR (20–60 ms following the onset of the stimulus), which is considered to be more difficult to encode relative to the steady-state (60–170 ms), or the vowel portion (White-Schwoch et al., 2017^9^). Whereas the steady-state consists of the same frequency cues over a larger interval of time, the formant transition necessitates accurate tracking of the speech formants over a shorter time window (Krizman & Kraus, 2019^10^). Another manipulation to evaluate FFR fidelity under adverse conditions was our use of the [da] FFR stimulus presented with background noise, which works to mask certain frequency and timing cues.

The discrepancy between Non-synchronizers and Synchronizers in terms of our FFR measures was especially apparent under certain conditions (i.e., steady-state, high frequency, and noise presentation mode). When those elements were present, Synchronizers experienced less degradation in their responses compared with their non-synchronizer peers. This dovetails with evidence on the importance of auditory processing in noise for literacy development. A model of development suggested by White-Schwoch et al., (2015^12^) presents reading difficulties as a function of impoverished auditory processing in early childhood. Previous studies have shown that children who are either poor readers or who have been diagnosed with some form of reading impairment (e.g., dyslexia) also have poor auditory processing of speech in noise (Chandrasekaran et al., 2009^19^; Hornickel et al., 2009^20^; Cunningham et al., 2001^21^; White-Schwoch et al., 2015^22^).

Our current study builds upon this foundation and adds that children who struggle with auditory–motor synchronization also have difficulties when encoding a noisy stimulus. Our findings support that the auditory system, which relies on the synchronous firing of subcortical circuits to pick up the components of language, has ties with motor circuits involving beat synchronization. The stimulus [da] noise places more pressure on the auditory system and allowed us to draw out systematic individual differences between the two synchronization groups. This enabled us to conclude that Synchronizers experience less degradation of the FFR in adverse listening conditions. In this way, both rhythmic drumming and the brain’s response to sound may provide windows into a child’s future success in reading.

Our study provided support also to previous evidence suggesting links between rhythm-related abilities and attention (Tierney & Kraus, 2013^5^; Khalil, Minces, McLoughlin, and Chiba, 2013^23^) by revealing how both attention and temporal processing are interlinked abilities with synchronization skills (Supplementary Fig. 1 and Supplementary Table 1. are focused on analyses investigating the role of attention in our data).

By establishing strong relationships between rhythm and language processes in a large group of typically developing preschoolers, our study motivates continued exploration of these relationships in a broader preschool population, including children at risk for or manifesting language disorders. Being able to assess these children in their beat synchronization skills and FFRs would provide us insights on how and when an atypical development could (or not) become manifest across both these domains.

Our study demonstrates how an auditory–motor synchronization task may serve as an important, early indicator of a child’s ability to understand spoken and written language. Based on our results that demonstrate a difference in preliteracy skills and auditory processing in children as young as 3 years old, we suggest that interventions, which may support these areas of development (e.g., music and rhythm training), be implemented early in childhood.

## METHODS

### Participants

One hundred and fifty-six children (70 females) between the ages of 3 and 5 years old (Mean = 4.02, SD = 0.61) were recruited from the Chicago area. On average, maternal education level is high (Mean = 5.48; SD = 1.22, range 1–7 with 1 = High school diploma and 7 = Doctorate degree). However, the participants are fairly diverse in terms of their home ZIP code’s median annual household income (Median min = $23,430; Median max = $248,240; 25th percentiles = $57,572 – 75th percentiles = $79,410). Children from Carr et al., 2014 and 2016 are included among our participants. The goal of the present study is to provide a comprehensive story considering all collected data. For transparency, we provide results in the supplementary material with the children in previous publications excluded (Supplementary Table 2.). All were monolingual-English speakers. None had a diagnosis of autism spectrum disorder, a history of neurologic conditions, or a family history of language-learning disorders. The children all passed a screening for peripheral auditory function (consisting of otoscopy, tympanometry, and distortion product otoacoustic emissions of at least 6 dB above the noise floor). In addition, click-evoked auditory brainstem responses to a 100 μs square-wave click stimulus presented at 80 dB sound-pressure level (SPL) in rarefaction at a rate of 31.3/s revealed normal auditory response timing (wave V latency < 5.84 ms, with Mean = 5.62 and SD = 0.144). Written informed assent and consent were obtained from the children and their legal guardians, and the children were monetarily compensated. All procedures were approved by the Northwestern University Institutional Review Board.

### Beat synchronization task

Beat synchronization was measured as a child’s performance on a drumming task. This type of measure was selected based on a previous study showing that children, especially those in the preschool age range, are better able to synchronize their body movements when the beat was provided by an actual experimenter and not just through a speaker (Kirschner & Tomasello, 2009^24^). Following the protocol by Kirschner and Tomasello, the experimenter and child sat across from one another, each with their own conga drum in front of them. The experimenter wore an in-ear headphone that delivered isochronous beats, which the experimenter would then replicate on the drum. The child was encouraged to drum along with the beats set out by the experimenter, who previously had to demonstrate the ability to reliably produce synchronous rhythms following a pacing tone (Supplementary Table 3 reports Rayleigh’s *p*-values for each experimenter across both ISI. All *p*-values are greatly below .001). Each of the child’s hits on the conga drum was recorded by a Pulse Percussion DR-1 trigger that sent this information as a voltage value to the recording computer, which saved these files using Audacity (version 2.0.5). Two rates of drumming were set by the experimenter for the child to replicate: 400 ms interstimulus interval (ISI), which equates to a rate of 150 beats/min, and 600 ms ISI, which is 100 beats/min (Kirschner & Tomasello, 2009^24^). Both of these rates were recorded with two trials each, for a total of four trials. Each trial lasted for 20 s, and the experimenter performed 50 hits for the 400 ms ISI and 33 hits for the 600 ms ISI rates.

Each participant’s Audacity file was processed in MatLAB (Mathworks, Inc., Natick, MA). Because each child drummed at different amplitudes and required a different refractory period (i.e. the length of time required for a drum hit to “die out”) thresholds had to be individually calculated for each participant. An increase in loudness past the amplitude threshold marked a hit, and a refractory period was placed immediately after the hit so that no hits would be marked during this pause. Each file was double-checked visually for missed or falsely identified drum hits after the computer processing.

**Fig. 8:**
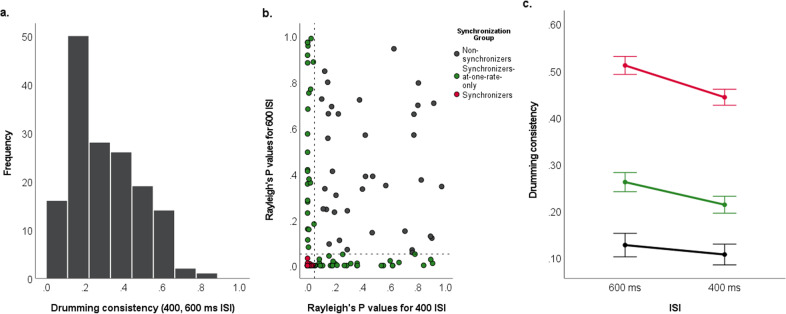
Distribution of drumming consistency (*R*-value) averaged between two rates of drumming, 400 and 600 ms ISI, from the beat synchronization task (*N* = 156). (**a**) Histogram of averaged measures of drumming consistency from all participants in the study. (**b**) Rayleigh’s *P* value for 400 ms ISI plotted against Rayleigh’s *P* value for 600 ms ISI. Vertical and horizontal lines at a *p*-value equal to 0.05 show the cutoff for categorization of “Synchronizer” (*p* < 0.05). (**c**) Line plot of drumming consistency across the two rates (600 and 400 ms) for each synchronization ability group (Error bars at ± 1 standard error).

From the drumming task, a measure for drumming consistency was calculated using circular statistics to convert hits into quantifiable vectors. From each hit on the conga drum by the child, a phase angle θ was calculated based on the difference between the time of the experimenter’s hit and the child’s hit, which was then divided by the ISI rate and multiplied by 360 degrees. This served to change the phase to a degree along a unit circle ranging from 0 to 360 degrees. We then summed all the vectors and divided the result by the number of drum hits produced, resulting in a mean vector R. The angle of this vector represents the extent to which the subject tended to lead or follow the stimulus hits, and the length of the vector is a measurement of the extent to which participants tended to maintain a constant temporal relationship between their drum hits and the stimulus hits—i.e., the extent to which they synchronized. The length of vector R was computed by averaging the synchronicity of the participant’s taps at each of the two trials across both drumming rates. Rayleigh’s test, which tests the consistency in the phase of the responses versus a uniform distribution around the circle, was then performed on the set of all of the vectors produced in the two trials for a given rate to determine whether a participant was significantly synchronizing to a stimulus. If a child’s Rayleigh’s test resulted in a P value of less than 0.05 at both rates, the child was classified as “Synchronizer”. If the *P* value was greater than 0.05 at both rates, the child was categorized as a “Non-synchronizer”. If the *P* value was less than 0.05 at one rate and greater than 0.05 at another rate, the child was classified as “Synchronizer-at-only-one-rate”. P values for individuals at each rate are detailed in Fig. 8b.

For all of the participants (*N* = 156), an average score of drumming consistency between the two rates (400 and 600 ms ISI) was calculated from the beat synchronization task. The distribution of scores and *p*-values from the task is shown in Fig. 8a. Based on the distribution shown in Fig. 8b and the positive whole-group age correlation (*r*_(154)_ = 0.200, *p* = 0.012), we decided to proceed with the main statistical analysis of this study by considering only the children who performed as “Synchronizers” at both rates (*N* = 63, 35 females) and those who were “Non-synchronizers” at both rates (*N* = 37, 9 females). This choice was motivated by the fact that these two groups can more reliably be classified as good or poor, respectively, with respect to beat synchronization. The group in the middle, “Synchronizers-at-one-rate-only”, is instead more difficult to classify because a difference in a level of performance between drumming rates could indicate that the child is having attentional difficulties during the beat synchronization assessment, he/she is becoming fatigued over the four trials of the task, or he/she has an actual tempo preference that impacts the synchronization performance (Fig. 8c). In order to provide a comprehensive picture, we devote a section in the “Results” showing where this third group fits with respect to all the behavioral and electrophysiological variables considered.

No significant group differences were found when limiting to “Non-synchronizers” and “Synchronizers” in terms of age (*F*_(1,99)_ = 3.234, *p* = 0.075, η*p*^2^ = 0.032), verbal IQ (*F*_(1,97)_ = 3.483, *p* = 0.065, η*p*^2^ = 0.035), nonverbal IQ (*F*_(1,97)_ = 2.894, *p* = 0.092, η*p*^2^ = 0.029), or click V ABR latency (F_(1,94)_ = 0.964, p = .329, ηp^2^ = .010). However, the two synchronization groups did differ in terms of sex. Rhythm performance based on drumming consistency was significantly different between the females and males (*F*_(1,99)_ = 9.962, *p* = 0.002, η*p*^2^ = 0.092). Furthermore, fewer females were categorized as Non-synchronizer (χ^2^ = 16.53, p = 0.002). Because of these differences, sex was controlled for in all the analyses performed.

### Verbal and nonverbal intelligence

IQ scores were assessed using the Wechsler Preschool and Primary Scale of Intelligence, 3rd edition (Pearson/PsychCorp), which tests cognitive development. Verbal intelligence was based on the raw scores in the information subtest. Nonverbal intelligence was measured using the raw scores from the object assembly subtest if the participant was between the ages of 3 and 4 years old or the matrix reasoning subtest if they were above the age of 4.

### Phonological awareness

The Phonological Awareness subtest was taken from the Clinical Evaluation of Language Fundamentals Preschool, second edition (CELF-P2; Pearson/PsychCorp). This subtest evaluates the child’s ability to identify certain sounds or phonemes within words and produce rhyming words. Using these raw scores, we are able to gain a general sense of each child’s basic knowledge of sound and language structures. This test was administered only to children aged 4 or older. Because of this restriction, we evaluated this particular preliteracy skill in a smaller subset of children-specifically, 108 out of the total 156 participants.

### Auditory short-term memory

Auditory short-term memory was taken from the Recalling Sentences subtest of CELF-P2 and was administered to all preschoolers. The child was asked to memorize and verbally repeat sentences that varied in terms of length and complexity. A raw score was given based on how closely the child was able to adhere exactly to the sentence that was presented to them, in line with that reported in the CELF-P2 manual (Administration and Scoring directions).

### Rapid automatized naming

Rapid automatized naming of objects (RAN-O) and colors (RAN-C) measured how quickly a child was able to recognize an object or color and name them out loud (Pro-Ed, Inc.). Prior to each recording, the experimenter made sure that the child was familiar with all the stimuli that would be presented and then administered both subtests, which each contained fifteen objects or colors. The experimenter recorded the time it took for the child to completely and accurately name all of the stimuli. A lower raw score indicates a faster response time, measured in milliseconds, which also means higher proficiency in RAN.

### Music perception

Each child’s ability to perceive differences in melody or rhythm in short musical phrases was evaluated using Gordon’s AUDIE musical perception assessment. In this task, the child was familiarized with a “special song,” which was a three-note musical phrase. The child was asked to remember this short series of notes and then asked to identify if the next notes played differed from the original in terms of melody or rhythm, depending on the subtest. Each subtest contained 10 questions. A d’ sensitivity score was calculated using signal detection theory. The AUDIE task was given to the participants for the purpose of testing whether the drumming task is a reliable measure for broader musical awareness.

### Frequency following response (FFR)

Stimuli were presented using E-Prime (Version 2.0; Psychology Software Tools, Inc.) via an earphone inserted into the right ear (ER-3, Etymotic Research).

The evoked responses were recorded with BioSEMI Active 2 (BioSEMI). Surface electrodes were placed on the skin such that the active electrode was on the vertex (Cz), reference electrodes on the fronts of both the right and left earlobes, and grounding electrodes at both sides of the forehead (Fp1 and Fp2). The sites of application were prepared using prepping gel (NUPrep^TM^) and secured with conductive paste (Ten20). Since the stimuli were presented into the right ear, the ipsilateral responses were analyzed, which includes data recorded differentially between the Cz and right earlobe electrodes. Because FFRs can be recorded during passive listening, the children sat in a comfortable chair while watching a movie of their choice (soundtrack of the movie set at less than 40 dB SPL as to not cover the sound of the stimulus) in a sound-proof booth.

Frequency-following responses (FFRs) were elicited from four different stimuli: [ba], [da], [ga], and [da] presented in a noisy condition. Each 170 ms stimulus consisted of a single syllable that was synthesized with a 5 ms duration-onset burst, followed by a formant transition through 60 ms, then a steady-state vowel from 60 to 170 ms. The frequencies for each stimulus are described as follows

*[ba]* – The [ba] stimulus has a second formant frequency of 900 Hz, which transitions to 1240 Hz during the vowel portion of the stimulus.

*[ga]* – The [ga] stimulus has a second formant frequency of 2480 Hz, which transitions to 1240 Hz during the vowel portion of the stimulus.

*[da]* – The [da] stimulus has a second formant frequency of 1700 Hz, which transitions to 1240 Hz during the vowel portion of the stimulus.

*[da]noise* – Similar presentation as the [da] stimulus but with a multi-talker background babble added at 10 dB less than the signal.

The presentation of the four stimuli was controlled by E-Prime 2.0 (Psychology Software Tools, Inc., Sharpsburg, PA, US). Each stimulus was presented with an 81 ms interstimulus interval (251 ms onset-to-onset) in alternating polarities, which entailed inverting the presented waveform by 180 deg on half of the trials. FFRs were recorded from both polarities, and the responses were then added together (added polarity) to derive an envelope-biased response or subtracted (subtracted polarity) to derive a response biased to the temporal fine structure (Krizman and Kraus, 2019^10^). Each syllable was presented 2100 times per polarity.

The responses were digitized at 16.384 kHz with an online band-pass filter of 100–3000 Hz (20 dB/decade roll-off) in the BioSEMI ActiABR module for LabView 2.0 (National Instruments, Austin, TX, US). The responses, per manufacturer specification, were offline-amplified in the frequency domain using custom software in MATLAB (*The Mathworks, Inc., Natick, MA, US)*. Responses were amplified 20 dB per decade for 3 decades below 100 Hz (0.1–100 Hz). Next responses were bandpass filtered to the frequency region of interest for the responses (70–2,000 Hz, Butterworth filter, 12 dB/octave roll-off, zero phase shift), epoched from −40–210 ms (stimulus onset at 0 ms), baselined, and artifact rejected (± 35 μV).

### Envelope-encoding precision

Envelope-encoding precision is calculated from cross-correlation between the envelopes of the waveform of the evoking stimulus and the participant’s frequency following response. The stimulus waveform was the first bandpass filtered to align with the neural response (at 70–2000 Hz) with a 12 dB per octave roll-off. Next, Hilbert transforms were performed on the stimulus and response waveforms. Finally, a 200 Hz low-pass filter was applied to dampen the high frequencies and pull out the low-frequency envelope (the 200 Hz low-pass filter was applied only to envelope-based analysis). To calculate the precision between the stimulus and the participant’s neural encoding of the envelope, the maximum of a cross-correlation function between the filtered Hilbert-transformed stimulus and response was obtained within a lag of 5 and 12 ms. This lag range accounts for the time required for the signal to traverse from the cochlea to the rostral brainstem. The correlation value was then z-transformed to be used for statistical analyses.

### Response consistency (RC)

Response consistency measures the stability of the neural response between trials and is represented by the correlation between two subaveraged waveforms from 0 to 170 ms. From the total number of sweeps, two subaveraged waveforms (2000 sweeps each) were generated by random sampling. This random generation was done 300 times. The final value for response consistency is the average of those 300 z-transformed correlation values.

### Phase-locking consistency (PLC)

Phase-locking consistency represents the variability in the timing of the frequency encoding in the response to each stimulus. This measure was calculated in 20 Hz windows surrounding the fundamental frequency of the stimulus (100 Hz) and its harmonics up to 1000 Hz. Time-frequency spectrum was calculated using a short-time fast Fourier transform that resulted in a matrix containing two measures for each time x frequency point: a vector length (the extent to which each frequency is encoded in the FFR) and phase (the timing of that frequency). To specifically analyze the timing variability, each vector was transformed into a unit vector. For each frequency, the 4000 vectors were averaged, and the length of the resulting vector was calculated as a measure of the consistency of the firing time with respect to the stimulus and is the value for phase-locking consistency.

The averaged vector length ranges between 0 and 1, with a higher value indicating greater consistency. For this study, PLC was averaged across a low-frequency harmonic range (100–400 Hz for added polarity, 200–400 Hz for subtracted polarity) and a high frequency harmonic range (500–1000 Hz for both polarities).

The datasets generated during and/or analyzed during the current study are available from the corresponding author on reasonable request.

### Statistical analysis

Statistical analysis was performed with SPSS (SPSS, Inc., Chicago, IL).

Mean comparisons were used to evaluate the differences in performance on neuropsychological, and neural measures between “Non-synchronizers” and “Synchronizers”. Sex was included as a covariate given that drumming consistency differs between males and females (see “Methods” section for more details).

One-way ANOVAs were used to compare group performance differences on the selection of neuropsychological assessment measures, phonological awareness, auditory short-term memory, rapid automatized naming, and music perception.

Repeated-measure ANOVA’s were used to compare mean differences across the neural measures-envelope precision, RC, and PLC. The synchronization ability groups, “Non-synchronizers” and “Synchronizers”, were used as *Between-group* variable. The *Within-subjects* factors include the following aspects of the FFR, depending on the specific FFR measure:

- Time region of the response:

1. Formant transition (from the consonant onset at 20 ms to the vowel at 60 ms)
2. Steady-state vowel (from 60 ms to the offset of the stimulus at 170 ms)

- Presentation mode:

1. Quiet (the quiet speech stimuli were averaged together to represent one, composite quiet FFR stimulus. Supplementary Figure 1 in the supplementary material illustrates similarities between the Quiet stimuli for each FFR measure)
2. Noise

- Polarity:

1. Added
2. Subtracted

- Frequency range:

1. Low frequencies (100–400 Hz) (for subtracted polarity, low frequencies were computed from 200 to 400 Hz)
2. High frequencies (500–1000 Hz)

For Envelope-encoding precision, the within-subject factors considered are (1) time region of the response and (2) presentation mode

For Response consistency, the within-subject factors considered are (1) time region of the response, (2) presentation mode, and (3) polarity

For Phase-locking consistency, the within-subject factors considered are (1) time region of the response, (2) presentation mode, (3) polarity, and (4) frequency range.

These comparisons were then analyzed using additional *post hoc* independent sample *t*-tests.

### Reporting summary

Further information on research design is available in the Nature Research Reporting Summary linked to this article.

## Supplementary information

Supplementary Information

Reporting Summary

## Data Availability

The datasets generated during and/or analyzed during the current study are available from the corresponding author on reasonable request.
