# Supplementary material for: Rhythm, reading, and sound processing in the brain in preschool children
Source: NPJ Sci Learn. 2021 Jun 29;6:20. doi: 10.1038/s41539-021-00097-5 (PMC8242059; doi:10.1038/s41539-021-00097-5)
Supplement: Supplementary file 1 — Supplementary Information [file 41539_2021_97_MOESM1_ESM.pdf]

Supplementary material

- Role of Attention

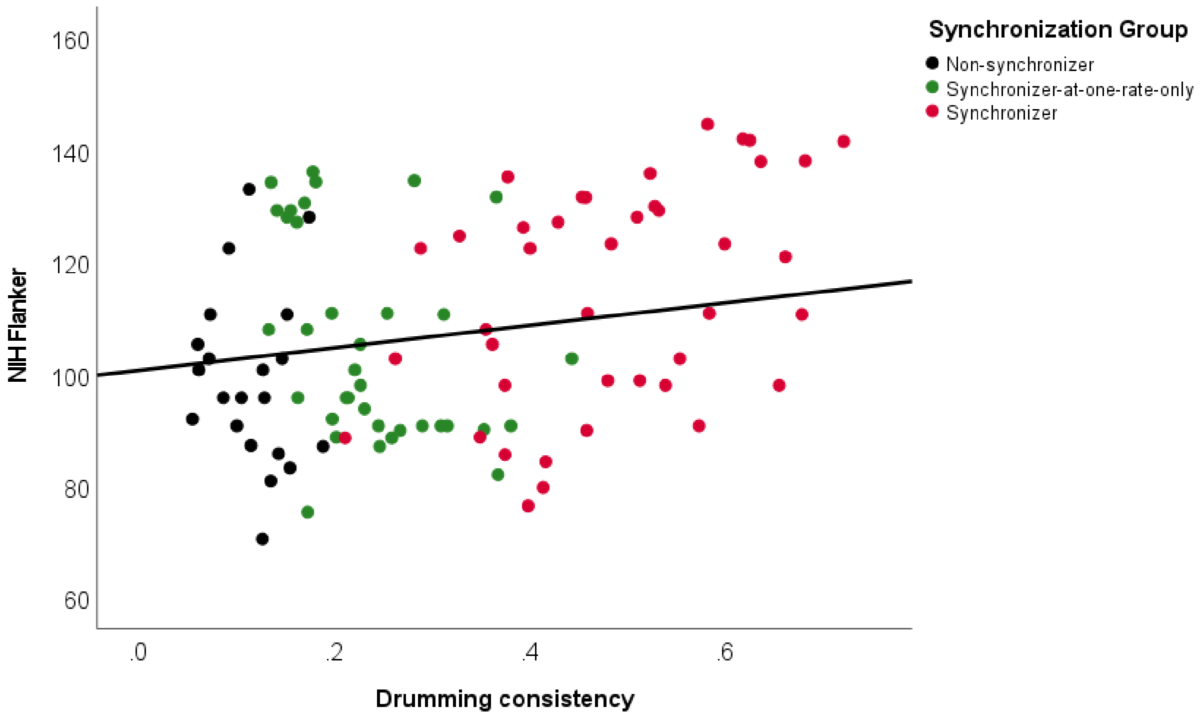

Supplementary Figure 1. Drumming consistency positively correlates with performance on NIH Flanker score.

Supplementary Table 1. **Comparison between statistical values when running RM ANOVA's with NIH Flanker performance as covariate and without it as reported in the main body of the manuscript for Envelope Encoding Precision, Response Consistency, and Phase Locking Consistency**

	With attention as covariate				All kids			
**Envelope Encoding Precision**	**F**	**df**	**p-value**	**np²**	**F**	**df**	**p-value**	**np²**
ME Group	9.022	51	.004	.150	10.860	87	.001	.111
ME Presentation mode	5.530	51	.023	.098	10.867	87	.001	.111
ME Time region	4.211	51	.045	.076	6.602	87	.012	.071
Group*Presentation mode*Time region	5.515	51	.023	.098	4.314	87	.041	.0
**Response Consistency**	**F**	**df**	**p-value**	**np²**	**F**	**df**	**p-value**	**np²**
ME Group	3.625	46	.063	.073	4.593	79	.035	.055
ME Presentation mode	6.844	46	.012	.130	12.545	79	.001	.137
ME Time region	.599	46	.443	.013	.879	79	.351	.011
ME Polarity	17.757	46	.000	.279	23.197	79	<.001	.227
Group*Time region	13.047	46	.001	.221	8.091	79	.006	.093
Group*Presentation mode*Polarity	5.589	46	.022	.108	9.860	79	.002	.111
Group*Time region*Polarity	11.698	46	.001	.203	4.316	79	.041	.052
Group*Presentation mode*Time region*Polarity	40.504	46	.000	.468	11.068	79	.001	.123
**Phase Locking Consistency**	**F**	**df**	**p-value**	**np²**	**F**	**df**	**p-value**	**np²**
ME Group	5.595	46	.022	.108	4.498	76	.037	.056
ME Presentation mode	2.295	46	.137	.048	8.927	76	.004	.105
ME Time region	19.234	46	.000	.295	22.238	76	<.000	.226
ME Polarity	.954	46	.334	.020	.559	76	.457	.007
ME Frequency	4.484	46	.040	.089	10.585	76	.002	.122
Group*Time region	17.068	46	.000	.271	9.182	76	.003	.108
Group*Time region*Presentation mode	4.604	46	.037	.091	5.217	76	.025	.064
Group*Time region*Frequency	7.548	46	.009	.141	7.037	76	.010	.085

- Exclusion participants in previous studies (Carr et al., 2014; 2016) - Behavioral and FFRs data

Supplementary Table 2. Comparison between statistic values when running ANOVA's excluding the kids in Carr et al., 2014* or 2016 and considering the entire dataset as reported in the main body of the manuscript.**

	Excluding kids from Carr et al., 2014* or 2016**				All kids			
**Behavioral measures***	**F**	**df**	**p-value**	**np²**	**F**	**df**	**p-value**	**np²**
Phonological Awareness	6.525	30	.016	.189	11.680	55	<.001	.175
Auditory short-term memory	1.910	60	.172	.032	4.773	91	.031	.050
Rapid automatized naming	3.744	61	.058	.060	4.881	88	.030	.053
Gordon music perception	.074	63	.787	.001	2.716	92	.103	.029
**Envelope Encoding Precision***	**F**	**df**	**p-value**	**np²**	**F**	**df**	**p-value**	**np²**
ME Group	7.632	54	.008	.124	10.860	87	.001	.111
ME Presentation mode	2.642	54	.110	.047	10.867	87	.001	.111
ME Time region	9.768	54	.003	.153	6.602	87	.012	.071
Group*Presentation mode*Time region	1.649	54	.205	.030	4.314	87	.041	.0
**Response consistency****	**F**	**df**	**p-value**	**np²**	**F**	**df**	**p-value**	**np²**
ME Group	7.635	55	.008	.122	4.593	79	.035	.055
ME Presentation mode	8.113	55	.006	.129	12.545	79	.001	.137
ME Time region	.164	55	.687	.003	.879	79	.351	.011
ME Polarity	11.911	55	.001	.178	23.197	79	<.001	.227
Group*Time region	8.998	55	.004	.141	8.091	79	.006	.093
Group*Presentation mode*Polarity	3.699	55	.060	.063	9.860	79	.002	.111
Group*Time region*Polarity	6.107	55	.017	.100	4.316	79	.041	.052
Group*Presentation mode*Time region*Polarity	7.519	55	.008	.120	11.068	79	.001	.123
**Phase locking consistency****	**F**	**df**	**p-value**	**np²**	**F**	**df**	**p-value**	**np²**
ME Group	8.284	52	.006	.137	4.498	76	.037	.056
ME Presentation mode	2.503	52	.120	.046	8.927	76	.004	.105
ME Time region	11.239	52	.001	.178	22.238	76	<.000	.226
ME Polarity	.005	52	.941	.000	.559	76	.457	.007
ME Frequency	8.350	52	.006	.138	10.585	76	.002	.122
Group*Time region	9.884	52	.003	.160	9.182	76	.003	.108

Group*Time region*Presentation mode	8.418	52	.005	.139	5.217	76	.025	.064
Group*Time region*Frequency	9.737	52	.003	.158	7.037	76	.010	.085

- Experimenter's synchronization skills

Supplementary Table 3. Rayleigh's p-values for each experimenter across both ISI. All p-values are greatly below .001 confirming excellent synchronization skills across all the nine experimenters involved in testing.

Experimenter	p-values 600 ms ISI	p-values 400 ms ISI
**1**	3.85228E-22	5.77136E-41
**2**	1.31845E-22	5.01308E-28
**3**	3.8485E-32	3.63706E-24
**4**	6.64401E-38	1.06233E-56
**5**	5.87151E-31	8.13726E-43
**6**	5.08816E-15	6.27791E-27
**7**	1.0601E-24	1.89721E-10
**8**	7.00256E-38	6.97806E-49
**9**	3.4791E-21	3.34907E-41

- Quiet stimuli and DaNoise stimulus

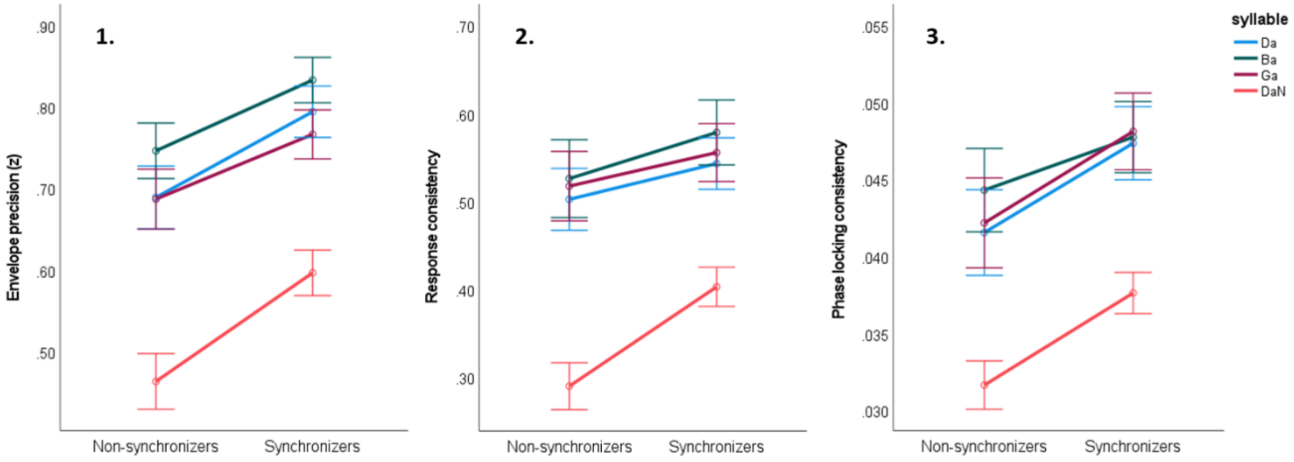

Supplementary Figure 2. Each subplot focuses on a specific FFR measure (1. Envelope precision, 2. Response consistency, 3. Phase locking consistency) and illustrates line plots for each stimulus considered (Da, Ba, Ga, DaN) across the two Synchronization groups (Non-synchronizers – Synchronizers). It is possible to appreciate the similarity between the Quiet stimuli (Da, Ba, Ga) versus the DaNoise stimulus that motivates our choice to compute composite measures across the Quiet stimuli for each FFR measure.
