# Supplementary material for: Rhythm, reading, and sound processing in the brain in preschool children
Source: NPJ Sci Learn. 2021 Jun 29;6:20. doi: 10.1038/s41539-021-00097-5 (PMC8242059; doi:10.1038/s41539-021-00097-5)
Supplement: Supplementary file 2 — Reporting Summary [file 41539_2021_97_MOESM2_ESM.pdf]

nature research

Corresponding author(s): Nina Kruas
Last updated by author(s): 5/17/2021

# Reporting Summary

Nature Research wishes to improve the reproducibility of the work that we publish. This form provides structure for consistency and transparency in reporting. For further information on Nature Research policies, see our Editorial Policies and the Editorial Policy Checklist.

## Statistics

For all statistical analyses, confirm that the following items are present in the figure legend, table legend, main text, or Methods section.

| n/a | Confirmed | |
|---|---|---|
| ☐ | ☒ | The exact sample size (*n*) for each experimental group/condition, given as a discrete number and unit of measurement |
| ☐ | ☒ | A statement on whether measurements were taken from distinct samples or whether the same sample was measured repeatedly |
| ☐ | ☒ | The statistical test(s) used AND whether they are one- or two-sided<br>*Only common tests should be described solely by name; describe more complex techniques in the Methods section.* |
| ☐ | ☒ | A description of all covariates tested |
| ☐ | ☒ | A description of any assumptions or corrections, such as tests of normality and adjustment for multiple comparisons |
| ☐ | ☒ | A full description of the statistical parameters including central tendency (e.g. means) or other basic estimates (e.g. regression coefficient) AND variation (e.g. standard deviation) or associated estimates of uncertainty (e.g. confidence intervals) |
| ☐ | ☒ | For null hypothesis testing, the test statistic (e.g. *F*, *t*, *r*) with confidence intervals, effect sizes, degrees of freedom and *P* value noted<br>*Give P values as exact values whenever suitable.* |
| ☒ | ☐ | For Bayesian analysis, information on the choice of priors and Markov chain Monte Carlo settings |
| ☒ | ☐ | For hierarchical and complex designs, identification of the appropriate level for tests and full reporting of outcomes |
| ☐ | ☒ | Estimates of effect sizes (e.g. Cohen's *d*, Pearson's *r*), indicating how they were calculated |

*Our web collection on statistics for biologists contains articles on many of the points above.*

## Software and code

Policy information about availability of computer code

| | |
|---|---|
| Data collection | Sound (drumming) recordings: Open source Audacity (v. 2.0.5) sound editing software was used to collect the sound samples.<br>EEG (FFR) recordings: A BioSemi Active 2 system, controlled by software provided by the manufacturer based on the Labview platform, was used to collect EEG data. E-Prime software delivered the auditory stimuli. |
| Data analysis | Sound and FFR samples were analyzed in commercial Matlab (v. 2015A) software using custom-coded routines. |

For manuscripts utilizing custom algorithms or software that are central to the research but not yet described in published literature, software must be made available to editors and reviewers. We strongly encourage code deposition in a community repository (e.g. GitHub). See the Nature Research guidelines for submitting code & software for further information.

## Data

Policy information about availability of data

All manuscripts must include a data availability statement. This statement should provide the following information, where applicable:

- Accession codes, unique identifiers, or web links for publicly available datasets
- A list of figures that have associated raw data
- A description of any restrictions on data availability

The datasets generated during and/or analyzed during the current study are available from the corresponding author on reasonable request.

# Field-specific reporting

Please select the one below that is the best fit for your research. If you are not sure, read the appropriate sections before making your selection.

☐ Life sciences ☒ Behavioural & social sciences ☐ Ecological, evolutionary & environmental sciences

For a reference copy of the document with all sections, see nature.com/documents/nr-reporting-summary-flat.pdf

# Behavioural & social sciences study design

All studies must disclose on these points even when the disclosure is negative.

| | |
|---|---|
| Study description | This study includes mainly quantitative data. |
| Research sample | One hundred and fifty-six children (70 females) between the ages of 3 and 5 years old were recruited from the Chicago area. On average, maternal education level is high (Mean = 5.48; SD = 1.22, range 1 – 7 with 1 = High school diploma and 7 = Doctorate degree). The participants are fairly diverse in terms of their home ZIP code's median annual household income (Median min = $23,430; Median max = $248,240; 25th percentiles = $57,572 – 75th percentiles = $79,410). |
| Sampling strategy | The participants were included in the study in a rolling basis. |
| Data collection | Pen and paper were used to assess the children with standardized tests. Drumming was picked up by a drum trigger placed on the bottom of the drum head and recorded as an audio file using Audacity software. E-Prime and the BioSEMI Active 2 systems were used for FFR data collection. |
| Timing | The data collection for this study started in 2013 and continued up until 2017. This study is part of a larger longitudinal project. |
| Data exclusions | No data were excluded for this study |
| Non-participation | No participants declined to participate |
| Randomization | No randomization was necessary for this study |

# Reporting for specific materials, systems and methods

We require information from authors about some types of materials, experimental systems and methods used in many studies. Here, indicate whether each material, system or method listed is relevant to your study. If you are not sure if a list item applies to your research, read the appropriate section before selecting a response.

## Materials & experimental systems

| n/a | Involved in the study | |
|---|---|---|
| ☒ | ☐ | Antibodies |
| ☒ | ☐ | Eukaryotic cell lines |
| ☒ | ☐ | Palaeontology and archaeology |
| ☒ | ☐ | Animals and other organisms |
| ☐ | ☒ | Human research participants |
| ☒ | ☐ | Clinical data |
| ☒ | ☐ | Dual use research of concern |

## Methods

| n/a | Involved in the study | |
|---|---|---|
| ☒ | ☐ | ChIP-seq |
| ☒ | ☐ | Flow cytometry |
| ☒ | ☐ | MRI-based neuroimaging |

## Human research participants

Policy information about studies involving human research participants

| | |
|---|---|
| Population characteristics | Please consider sections above for Age and Sex information.<br><br>All the participants monolingual-English speakers. None had a diagnosis of autism spectrum disorder, history of neurologic conditions, or a family history of language learning disorders. The children all passed a screening for peripheral auditory function (consisting of otoscopy, tympanometry, and distortion product otoacoustic emissions of at least 6 dB above the noise floor). In addition, click-evoked auditory brainstem responses to a 100-µs square-wave click stimulus presented at 80 dB sound pressure level (SPL) in rarefaction at a rate of 31.3/s revealed normal auditory response timing (wave V latency < 5.84 ms, with Mean = 5.62 and SD = 0.144). |
| Recruitment | Participants were recruited using flyers posted in local preschools and libraries. Some recruitment fairs were also arranged. |

| Ethics oversight | Northwestern University Institutional Review Board |
|---|---|

Note that full information on the approval of the study protocol must also be provided in the manuscript.
